# Subclavian Artery Pseudoaneurysm in an Eight-Year-Old Boy: A Rare Case Report and Review of Literature

**DOI:** 10.7759/cureus.41488

**Published:** 2023-07-07

**Authors:** Ogbonnaya Akuma, Chinaza Akuma, Lokeshwar Raaju Addi Palle, Carlo Kristian C Carredo, Subhan Savul, Shehryar Khawer, Aadil Khan

**Affiliations:** 1 Internal Medicine, Ebonyi State University, Abakaliki, NGA; 2 Public Health, Chamberlain University, College of Health Professions, Chicago, USA; 3 Department of Surgery, Kamala Children's Hospital, Chennai, IND; 4 General Surgery, Cebu Institute of Medicine, Cebu, PHL; 5 Internal Medicine, Ziauddin University, Karachi, PAK; 6 Department of Internal Medicine, Lala Lajpat Rai Hospital, Kanpur, IND

**Keywords:** computed tomography (ct) angiogram, us-guided, subclavian artery, embolization, arterial pseudoaneurysms

## Abstract

Pseudoaneurysms, also known as fake aneurysms, are balloon-like bulges that develop in the arteries and veins. This can happen due to injury, surgery, infection, or other conditions that damage blood vessels. Pseudoaneurysms are usually asymptomatic but can bleed and be painful. Left untreated, they can lead to severe complications such as thrombus formation and distant embolization. Subclavian pseudoaneurysms are rare, which can lead to potentially life-threatening complications of traumatic or iatrogenic injuries to the subclavian artery, such as catheterization. Prompt diagnosis and management are essential to avoid devastating outcomes. We report the case of a pediatric patient who developed a subclavian pseudoaneurysm after neck trauma and was successfully treated with endovascular embolization. This case highlights the importance of timely management and vigilant monitoring for this rare but potentially life-threatening condition.

## Introduction

In the pediatric population, subclavian artery pseudoaneurysms following trauma are infrequently reported. These lesions can be fatal, mainly when they manifest with bleeding or airway compromise, and can also cause significant morbidity by compressing over the adjacent vascular structures and brachial plexus [1]. A number of radiological interventions, such as ultrasound (US)-guided compression, percutaneous glue or thrombin injection, and endovascular procedures like embolization and stenting, have developed as therapeutic options from the traditional surgical approach toward a minimally invasive procedure in the early decades, significantly lowering the morbidity and mortality rates [2]. We report the case of a pediatric patient who developed a subclavian pseudoaneurysm after neck trauma. The patient was successfully treated with endovascular embolization.

## Case presentation

An eight-year-old boy was brought to the surgical outpatient department with swelling on the right side of his neck for the last 17 days with a history of trivial injury when a classmate hit him with a sharp pencil on the root of the neck just above the right clavicle. Parents reported a minor bleed which stopped after applying pressure. The swelling started appearing and gradually enlarged. He was taken to a general physician who examined his neck; the swelling was non-tender and a palpable thrill with audible bruit was noticed. He was treated based on post-traumatic inflammation and swelling in his right neck. His past medical history and family history were insignificant. On examination, he was conscious and well oriented, and physical examination revealed patient airways, no cornage nor triage were present, he was breathing normally, not complaining about dyspnea, his respiratory rate was 20 breaths per minute, the trachea was lying on the midline, there were no jugular veins turgor, vesicular murmur was bilaterally present and symmetric; a chest plain radiography was performed, there were no sign of pneumothorax. The patient was hemodynamically stable, the skin was warm and dry, blood pressure was 120/90 mmHg with a 100/minute heart rate, and he was resuscitated with 500 ml of isotonic physiologic solution. There was no subcutaneous crepitation.

**Figure 1:**
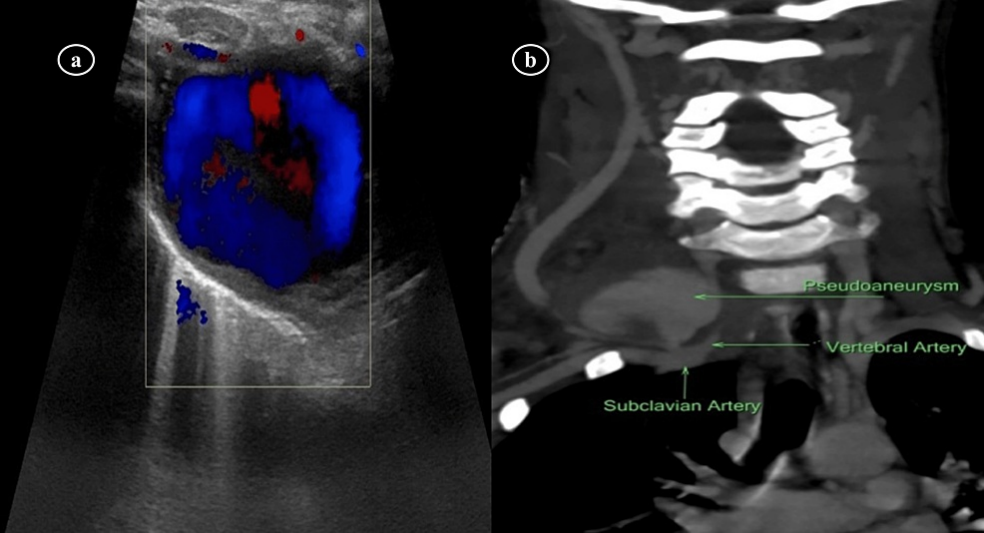
Color Doppler ultrasonography of the neck Color Doppler of the neck demonstrating the cystic structure of approximately 3 x 3 cm with the color flow and typical swirling motion called the “yin-yang” sign (a) suggestive of pseudoaneurysm arising from the right subclavian artery, and CT angiogram showing no associated arterio-venous fistula (b).

Doppler ultrasonography (USG) of the neck was performed, which showed a hypoechoic cystic structure of approximately 3 x 3 cm with the color flow within it showing a typical swirling motion called the “yin-yang” sign, as shown in Figure 1a, and a diagnosis of a narrow neck pseudoaneurysm arising from the right subclavian artery was established. A computed tomography (CT) angiogram and venogram of the neck vessels were then performed to delineate the exact anatomy and to rule out any associated arterio-venous fistula, as shown in Figure 1b.

After consultation with the interventional vascular radiology team, compression of the neck of the pseudoaneurysm was attempted under USG guidance, but no thrombosis was attained after two attempts of 15 minutes each. As a result, a plan for percutaneous injection of thrombin was made, and 1 ml of thrombin was injected under ultrasound guidance, and the lesion showed complete thrombosis post-procedure. A subsequent Doppler evaluation showed no flow within the pseudoaneurysm, and the flow in subclavian and distal vessels was maintained. The patient was observed for 30 minutes, and a repeated Doppler ultrasound showed complete thrombosis of the pseudoaneurysm.

**Figure 2:**
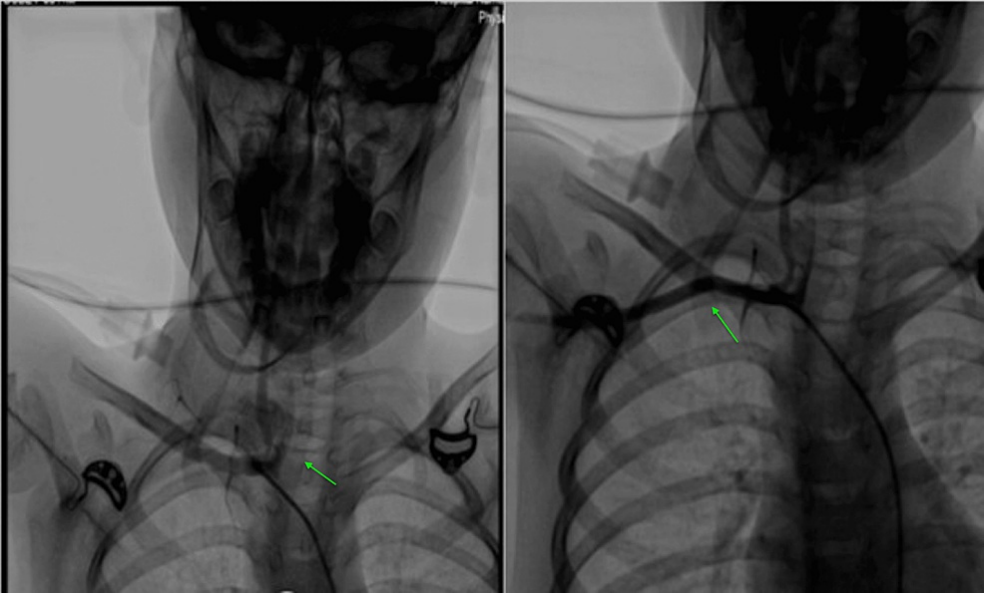
Intraarterial embolization Intraarterial embolization demonstrating pseudoaneurysm (arrow) and cannulation of the subclavian artery to look for its relations with the major artery (arrow).

Another Doppler ultrasound was done the next day, which showed partial refilling of the pseudoaneurysm sac with a thrombosed wall. Upon these findings, a plan for balloon-assisted glue injection was made. An intra-arterial embolization angiography Imaging showed pseudoaneurysm and cannulation of the subclavian artery to look for its relations with the major artery was performed, which revealed vertebral and left carotid artery in proximity with the aneurysmal sac as shown in Figure 2. An attempt to balloon-occlude the pseudoaneurysm would have resulted in occlusion of the carotid artery. However, attempts to cannulate the pseudoaneurysm directly failed.

**Figure 3:**
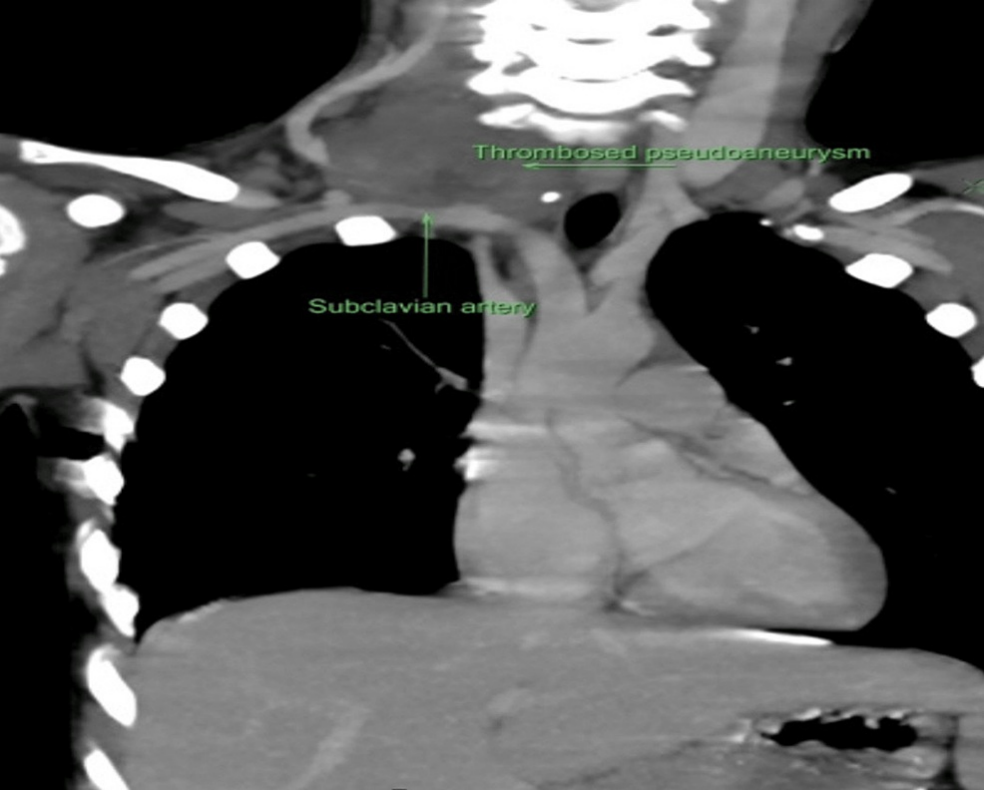
CT showing resolution of subclavian artery pseudoaneurysm

A reattempt of percutaneous thrombin injection was planned. Under direct ultrasound guidance, pseudoaneurysm was punctured, thrombin was injected, and complete sac thrombosis was seen post-procedure. Compression over the subclavian artery was maintained for 20 minutes post-procedure with Doppler visualization of the carotid artery so that flow within it could be monitored during the procedure. Repeated Doppler post-procedure 24 hours after the procedure did not show any refilling of the pseudoaneurysm, and a contrast CT scan was done before discharging the patient, which showed resolution as shown in Figure 3. The child was discharged the next day and followed up.

## Discussion

Trauma, inflammation, and iatrogenic procedures, such as central line placement, percutaneous drainage, or biopsy procedures, can cause injury to underlying vessels, leading to the formation of pseudoaneurysms. These pseudoaneurysms can dissect the blood into the surrounding tissues of the injured vessel, creating a blood-filled reservoir that communicates with the arterial lumen due to the arterial pressure [3]. Although conventional angiography remains the diagnostic standard of reference, Alternative techniques like duplex Doppler sonography, CT angiography, and magnetic resonance (MR) angiography can be employed to detect and diagnose pseudoaneurysms without invasive procedures. However, the accuracy of these methods may vary [4].

Because of its quick acquisition time (less than or equal to 1 minute) and minimal reliance on operator abilities, CT angiography offers benefits over other radiological interventions such as ultrasonography (USG), MR imaging, and angiography. Although initial postprocessing data to produce three-dimensional pictures takes time, axial images may provide diagnostic information necessary for surgical planning. Furthermore, a CT scan aids in detecting related injuries that may not be noticed by other modalities (for example, angiography, which is confined to evaluating arterial structures) [5]. The ability of gold-standard conventional angiography to perform the concurrent hemodynamic evaluation of a specific vascular channel, including identifying collateral vessels to determine donor artery expansion, is a significant advantage. Such evaluation is essential for management planning [6]. Pseudoaneurysms that cause intermittent or chronic bleeding should be addressed. However, because of the ambiguous and diverse natural history of pseudoaneurysms, especially when considering the anatomic placements of individual pseudoaneurysms, the clinical environment, and patient-associated conditions, the choice to treat asymptomatic pseudoaneurysms is debatable. Small, asymptomatic pseudoaneurysms should be monitored and treated only if they increase, do not resolve, or become symptomatic. However, no method exists to forecast which pseudoaneurysms will bleed and which thrombose spontaneously. As a result, certain specialists argue that decisive therapy should be performed in all such situations [7]. Initially, US-guided compression and thrombin injection can treat arterial pseudoaneurysms. However, with the advancement of minimally invasive therapeutic interventions, the associated morbidity and mortality rates have been massively reduced over the past decade and the cost of thrombin in such cases becomes a major limitation [4].

Ultrasound-guided compression and thrombin injection

A prospective study reported using ultrasound-guided compression (USGC) for treating pseudoaneurysms [7]. The success rate of this procedure ranges from 74% to 95% [8-10]. Although USDC is well-tolerated and cost-effective, being minimally invasive, it has been reported to be less likely successful in patients with a wider neck, using anticoagulation, or the pseudoaneurysm is large, long-standing, and multiloculated with the high-pressure flow. Arterial thrombosis or distal embolization are potential complications of the procedure. In patients with multiple surrounding injuries, infection, or overlying ischemia, USGC is not recommended [8].

The treatment for most post-traumatic or iatrogenic pseudoaneurysms is ultrasound-guided thrombin injections [9]. Thrombin injection into the pseudoaneurysm is based on the principle that thrombin plays a crucial role in converting fibrinogen to fibrin, resulting in the formation of a fibrin clot immediately after thrombin injection, even in the presence of antiplatelet or anticoagulation therapy. Extensive studies report success rates ranging from 91% to 100% [10]. In a study, failure to thrombose was linked to a size greater than 1.8 cm and concomitant use of anticoagulation or antiplatelet agents. While the exact rate remains unknown, the risk of spontaneous rupture of pseudoaneurysms increases with a size exceeding 3 cm, the presence of symptoms, large hematoma, or continued sac growth [11-14]. After injection, bed rest is unnecessary, but avoiding strenuous activity for 24 hours is recommended. Most asymptomatic patients are treated as outpatients and ambulated immediately after the procedure. When imaging is performed the following day, the recurrence rate ranges from 0% to 9% [12]. A follow-up duplex ultrasound is typically conducted within 24 to 72 hours after thrombin injection to monitor for recurrence. If a recurrence of the pseudoaneurysm is observed, it can be treated with additional injections (up to three), which have been shown to have excellent results [13].

Deep venous thrombosis, pulmonary embolism, and arterial thrombosis are the most severe complications associated with thrombin injection. Other patients have been observed while anticoagulated and reported spontaneous resolution without significant clinical sequelae [14]. It is important to note that thrombin is an off-label use for treating pseudoaneurysms, as the thrombin package warns that it is intended for topical use only, not for injection. Allergic reactions and anaphylaxis have been reported in patients previously exposed to bovine thrombin. Some experts recommend skin testing for patients with prior exposure to bovine thrombin, but this is not a common practice in most reports, and a skin test is rarely performed [15].

Percutaneous thrombin injection with balloon protection

This strategy aims to stop the pseudoaneurysm from leaking thrombin or thrombus into the original vessel [16]. The angiography suite is where the procedure takes place. A balloon catheter sized for the artery is put across the neck of the pseudoaneurysm after the artery is catheterized, and a catheter is inserted into the affected vessel across the pseudoaneurysm. A needle is percutaneously inserted into the pseudoaneurysm with Doppler ultrasonography. When the pulsatile flow in the balloon sac stops, the balloon has been positioned correctly and has received an acceptable amount of inflation [17]. Thrombin is administered once the flow into the pseudoaneurysm ceases. It takes ten minutes to inflate the balloon. After the balloon has been deflated, an angiogram and an ultrasound are done to check for complete thrombosis of the pseudoaneurysm. The process is repeated if a steady flow can be seen [18]. If the pseudoaneurysm neck is narrow, catheter-directed administration of coils into the sac is the recommended embolization method. Catheter-directed embolization materials may cure pseudoaneurysms with broad necks. Stent-graft (covered stent) insertion across the neck may eliminate wide-neck pseudoaneurysms. The possibility of stent-graft infection makes this approach unsuitable for mycotic pseudoaneurysms. Surgery is advised if the pseudoaneurysm is rapidly expanding and in danger of rupture, limb ischemia or distal emboli, significant soft tissue injury, infection, or previous treatments have failed [19].

## Conclusions

A large or expanding pseudoaneurysm should always be treated, and thrombin-induced percutaneous embolization with or without balloon occlusion of the native vessel is an easy procedure in trained hands with minimal or no chances of complications. Repeat attempts may be required sometimes, as in our case. Compression of the aneurysmal neck post-thrombin injection has not been advised in the literature. Still, we did a USG-guided neck compression in our second attempt, expecting it to help further stabilize the thrombus. However, it would be challenging to advocate it in all cases as no compression was required in most cases of pseudoaneurysm in children.
